# Blood Pressure Target in Acute Brain Injury

**DOI:** 10.5005/jp-journals-10071-23191

**Published:** 2019-06

**Authors:** Vivek Jain, Jitendra Choudhary, Rahul Pandit

**Affiliations:** 1-3 Department of Critical Care Medicine, Fortis Hospital, Mulund, Mumbai, Maharashtra, India

## Abstract

**How to cite this article:** Jain V, Choudhary J, Pandit R. Blood Pressure Target in Acute Brain Injury. Indian J Crit Care Med 2019;23(Suppl 2):S136–S139.

## INTRODUCTION

Both high and low blood pressure can cause organ dysfunction. Management of blood pressure (BP) is an important part of management of acute brain injury, as this protects the brain from secondary brain injury. Despite recent recommendation regarding blood pressure management, individualized BP and cerebral perfusion pressure (CPP) management remains challenging. This chapter attempts to answer different blood pressure targets in various acute brain injury conditions on the basis of recent evidence.

## CEREBROVASCULAR PATHOPHYSIOLOGY^1-3^

The general formula for cerebral blood flow (CBF) is as follows:

- CBF = (MAP – ICP) / CVR
- MAP- Mean arterial pressure, CVR= Cerebral vascular resistance
- CPP = MAP – ICP or CVP (whichever is higher) (ICP – Intracranial pressure, CVP- Central venous pressure).

Since neurons are highly dependent on adequate substrate delivery in order to maintain viability, cerebral blood flow (CBF) is tightly controlled. The brain maintains a constant CBF at approximately 50 mL/100 g/minute despite large changes in BP and CPP. When BP increases, cerebral vessels constrict and CVR increases in order to maintain a constant flow. When BP decreases or ICP increases, the vessels dilate and CVR decreases to keep CBF constant. The cerebral autoregulation is effective in a mean arterial pressure (MAP) range of 50–150 mm Hg in normotensive individuals. The relationship of CBF with systemic arterial pressure remains essentially nonlinear. This is to say the CBF depends on ABP, but does not increase or decrease in proportion to the increase or decrease in ABP. This nonlinear relationship between ABP and CBF is due to several associated factors, like autoregulation of the intracerebral blood vessels, and an intricate relationship between cardiovascular and cerebral vascular systems, ICP, autonomic nervous system and neurohumoral transmitters (Fig. 1).

When BP falls below the lower limit of autoregulation, CBF becomes completely dependent on CPP and thus, systemic blood pressure. The CBF, in that case, becomes compromised.

**Fig. 2:**
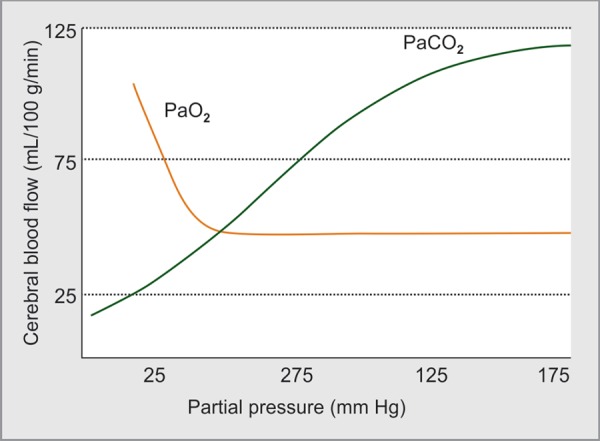
Effect of PaCO_2_ on CBF

The three main systems which control CBF are:

1. *Cardiovascular system*: The CBF depends on the systemic blood pressure which in turn depends on cardiac output as well as systemic vascular resistance. CBF is again related to cardiac output independent of the systemic blood pressure. Although, as already pointed out, the relationship between systemic arterial pressure and CBF remains nonlinear.
2. *Intracranial pressure*: As the skull is a rigid structure any rise in the pressure in any of the compartments of the brain, like vascular (raised ABP, neck position), cerebrospinal fluid (hydrocephalus) or brain parenchyma (trauma, ICH, space occupying lesion), will raise the ICP and can decrease the CPP, which in turn will reduce CBF.
  **Fig. 1:**
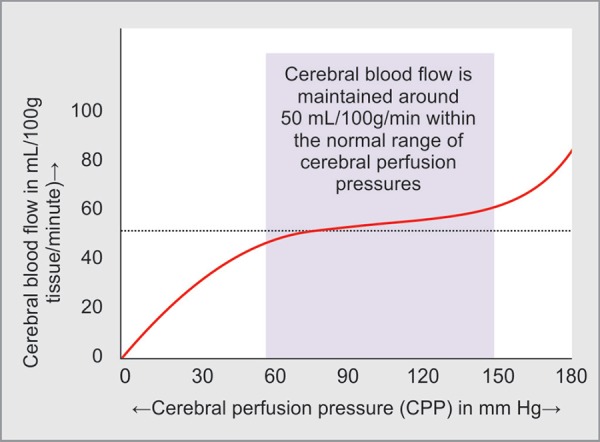
Cerebral blood flow and MAP
3. *Cerebral vascular system*: There is a considerable regulation of CVR, mainly at the level of intracerebral arterioles, but also at the level of arteries, veins, and capillaries. This autoregulation allows the brain to maintain satisfactory CBF, over a wide range of ABP. The cerebral autoregulation is mediated by mediators which may be carried by blood (like carbon dioxide, Fig. 2), produced locally at synapses or released by autonomic nerves. Dynamic cerebral autoregulation is the physiologic process that maintains CBF relatively constant in the face of beat to beat BP changes. Static cerebral autoregulation refers to CBF adjustments in response to more prolonged BP changes and is a measure of the overall efficiency of the system.

Normal cerebral cortical blood flow is 50 mL/100g tissue per minute. If this falls below 20 mL/100g/minute, there is impairment of the neuronal tissue, but still they remain salvageable. If the blood flow falls below 10 mL/100g/minute, there is irreparable damage to the neuronal tissues within a few minutes.

**Fig. 3:**
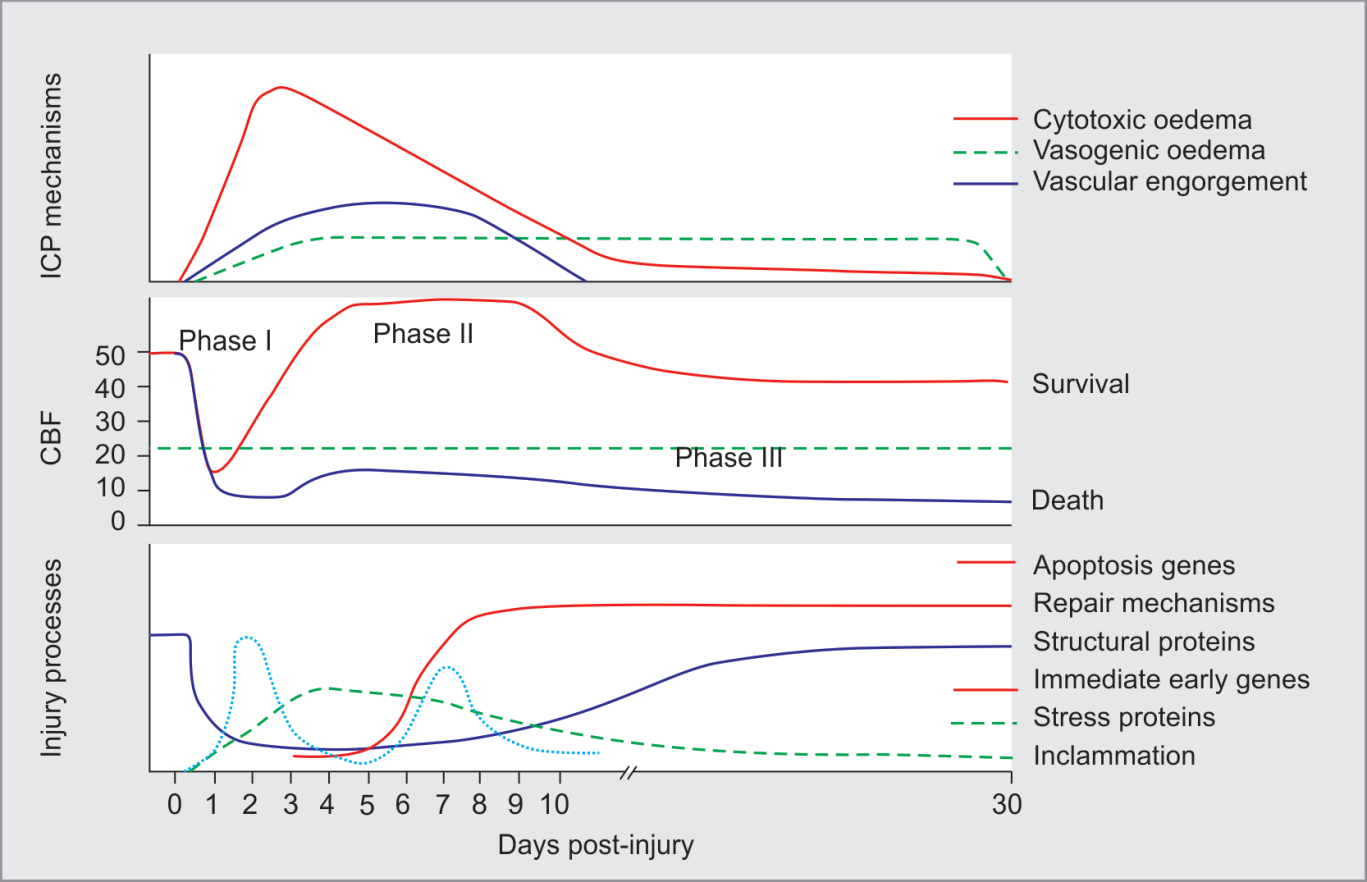
Patterns of CBF after TBI

Distinct patterns of cerebral blood flow have been described following head injuries that have direct clinical relevance with regard to management (Fig. 3).^4^

- *The hypoperfusion phase*: Cerebral blood flow is reduced by extrinsic and intrinsic mechanisms in the first 72 hours following injury, with resultant global and regional ischemia. Because autoregulation is impaired during this period, cerebral blood flow is largely dependent on systemic blood flow. Resultant neuronal ischemia may result in ‘cytotoxic’ cerebral edema and increased intracranial pressure.
- *The hyperemic phase*: Following the initial hypoperfusion phase, autoregulatory mechanisms may start to recover with improved cerebral blood flow. During this phase, intracranial inflammation and/or effects of medical therapies directed at maintaining adequate cerebral perfusion pressure may result in cerebral hyperemia and increased intracranial pressure. The consequences of hyperemia, inflammation and altered blood brain permeability may result in ‘vasogenic’ cerebral edema. This phase may persist for 7–10 days after injury in some patients.
- *The vasospastic phase*: This phase represents a complex of cerebral hypoperfusion due to arterial vasospasm, post traumatic hypometabolism and impaired autoregulation.

### Blood Pressure Management of Acute Brain Injury

Hypertension is a major risk factor for all strokes. Persistent elevation of BP in acute stroke can increase the size of hematoma in hemorrhagic stroke, cause edema with increased intracranial pressure at the site of stroke or cause hemorrhagic transformation of stroke. On the other hand, a rapid and substantial reduction in BP may reduce cerebral perfusion pressure and increase ischemic area. Lowering diastolic blood pressure was once the main target to achieve reduction of stroke and other cardiovascular event, but systolic blood pressure (SBP) has now become the target.

Although the role of longer-term BP control to improve outcomes in patients with stroke is undisputed, BP management immediately after a stroke remains controversial.

#### Blood Pressure Targets in Acute Ischemic Stroke (AIS)

Higher BP is common after acute ischemic stroke, especially in patients with history of chronic hypertension. ASA/AHA 2018 recommendations do not recommend ideal BP value for AIS patients.^6^ For patients undergoing thrombolytic therapy or endovascular intervention, it is recommended to lower BP to < 185/110 mm Hg at initiation of therapy and to maintain BP, 180/105 mm Hg for first 24 hours after intervention.

European Stoke Organization recommends to cautiously lower BP when BP is very high (>220/110 mm Hg) or in patients with severe cardiac failure, aortic dissection or hypertensive encephalopathy. Abrupt BP lowering should be avoided as per guidelines (initially lowering by 15%).^7^

*INVEST* ^5^ (Intravenous Nimodipine West European Stroke Trial suggest statistically significant deaths rates were associated with decrease in DBP >20% from baseline or DBP <60 mm Hg.

So we target for SBP <180 mm Hg in Acute ischemic stroke patients and we aim to achieve this target in first 6 hours of stroke and try to maintain this target for atleast 24 hours.

#### Blood Pressure Target in Acute Hemorrhagic Stroke

Elevated BP is associated with augmentation of hematoma size and this increase in hematoma corelates with worse prognosis. So recently there is great interest in BP manipulation to control hematoma and bleed size control. Various studies have tried to answer this question to various degrees.

*INTERACT 1* (Intensive Blood Pressure Reduction in Acute Cerebral Hemorrhage Trial)- shows that keeping SBP < 140 mm Hg significantly reduced hematoma progression compared to keeping BP <180 mm Hg. However, there was no difference in mortality or in stroke recurrence.^9^

Whereas in *INTERACT II* ^10^ results showed that larger SBP reductions in the acute phase of ICH are associated with the lower risk of a poor outcome, defined by the combination of death or disability.

Given these findings both ASA/AHA guidelines as well as European guidelines recommend that acute lowering of SBP to 140 mm Hg, when SBP is between 150 mm Hg and 220 mm Hg at presentation in ICH patients.^6,7^

ATACH 2 trial which was released recently compared aggressive BP control (SBP 110–139 mm Hg) vs standard care (SBP 140–179 mm Hg). Trial was terminated early for futility, there were no significant difference in outcome between 2 groups and in aggressive group incidence of renal adverse events were higher.^11^

Our pragmatic approach in ICH patients is to aim to control SBP at 140–160 mm Hg within 6 hours, using intravenous anti-hypertensives avoiding to rapid or to extreme BP reduction.

#### Blood Pressure Mangement in Traumatic Brain Injury (TBI) Patients

Systolic blood pressure plays a very important role in contributing secondary injury cascade after severe traumatic brain injury. As early as 1989, Klauber et al. reported a mortality of 35% in patient admitted with SBP <85 mm Hg, compared with only 6% in patients with a higher SBP.^9^ Additionally, hypotension has been shown to correlate with diffuse brain swelling.^10^ If autoregulation is not intact, there is dependency on SBP to prevent cerebral ischemia which is the single most important secondary insult.^12^

The 4th edition of BRAIN TRAUMA FOUNDATION recommends maintaining SBP >100 mm Hg for age 50–69 years (>110 mm Hg for age 15–49 years) is considered to decrease mortality and improve outcome. Though majority of guidelines target systolic BP, targeting cerebral perfusion pressure (CPP) is more physiological. Brain Trauma Foundation guidelines recommend routine CPP monitoring in severe TBI patients, which is said to decrease 2 weeks mortality. CPP target for survival and favourable outcome is between 60 mm Hg and 70 mm Hg.^13^

During initial phase of traumatic brain injury, till bleeding is not controlled one should aim for SBP target of >90 mm Hg. During hyperemic phase there is “luxury perfusion” because of increased cerebral blood flow due to vessel dilatation. During this phase one must target for CPP of around 60 mm Hg. Also, during late phase of TBI, there may be vasospastic phase, so you should be targeting slightly higher perfusion pressures by targeting higher SBP and MAP.

#### Blood Pressure Target in Subarachnoid Hemorrhage

Elevated BP is risk factor for rebleed in unsecured aneurysm. So, BP should be maintained less than 160 mm Hg as per ASA/AHA guidelines before aneurysm is clipped or coiled.^14^

Once aneurysm is secured secure induction of hypertension may be recommended for patients with delayed cerebral ischemia (DCI).

Our pragmatic approach is to maintain SBP <160 mm Hg till aneurysm is secured and then monitor for DCI. If patient at high risk of vasospasm or DCI then aim should be to maintain MAP >90 mm Hg or CPP >70 mm Hg.^14^

### Time to Treatment

In recent metanalysis, lowering BP in 15,432 patients did not change outcome in terms of stroke type, drug class or BP target used. However, there was tread toward less death or dependency and improved outcome, in patients who received treatment in first 6 hours after symptoms onset (INTERACT2, RIGHT). Larger BP changes seen in patients who were recruited early while small BP change seen in who were enrolled after 48 hours of onset. Subgroup analysis of ENOS (patients randomised to receive GTN within 6 hours) also showed reduced death or dependency and improved cognition and quality of life.^10,15,18^ Time is important factor to consider along with blood pressure targets. Early control of blood pressure in first few hours of stroke onset is important avenue which needs to look into.

## CONCLUSION

Despite various trials, there are no definitive recommendation regarding BP management targets in acute brain injury. BP management remains mainstay of treatment in acute brain injury patients. In early phase BP lowering may help in reducing hematoma progression and hemorrhagic transformation. In contrast BP management to avoid hypotension may be necessary to avoid secondary cerebral injuries. Various blood pressure targets along with timing of intervention and effect of different drugs is an important factor which needs to investigated to find out its effect of outcome.
